# PINOT NOIR: Pulmonic INsufficiency imprOvemenT with Nitric Oxide Inhalational Response

**DOI:** 10.1186/1532-429X-15-75

**Published:** 2013-09-04

**Authors:** Stephen A Hart, Ganesh P Devendra, Yuli Y Kim, Scott D Flamm, Vidyasagar Kalahasti, Janine Arruda, Esteban Walker, Thananya Boonyasirinant, Michael Bolen, Randolph Setser, Richard A Krasuski

**Affiliations:** 1 Cleveland Clinic Lerner College of Medicine of Case Western Reserve University, Cleveland, USA; 2 Hospital of the University of Pennsylvania and Children’s Hospital of Philadelphia, Philadelphia, USA; 3 Cleveland Clinic Imaging Institute, Cardiovascular Imaging, Cleveland, USA; 4 Cleveland Clinic Pediatric Institute, Pediatric Cardiology, Cleveland, USA; 5 Cleveland Clinic Quantitative Health Sciences, Cleveland, USA; 6 Cleveland Clinic Heart and Vascular Institute, Cardiovascular Medicine, Cleveland, USA

**Keywords:** Tetralogy of Fallot, Pulmonary insufficiency, Pulmonary regurgitation, Inhaled nitric oxide, Pulmonary vasodilation, Cardiovascular magnetic resonance

## Abstract

**Background:**

Tetralogy of Fallot (TOF) repair and pulmonary valvotomy for pulmonary stenosis (PS) lead to progressive pulmonary insufficiency (PI), right ventricular enlargement and dysfunction. This study assessed whether pulmonary regurgitant fraction measured by cardiovascular magnetic resonance (CMR) could be reduced with inhaled nitric oxide (iNO).

**Methods:**

Patients with at least moderate PI by echocardiography undergoing clinically indicated CMR were prospectively enrolled. Patients with residual hemodynamic lesions were excluded. Ventricular volume and blood flow sequences were obtained at baseline and during administration of 40 ppm iNO.

**Results:**

Sixteen patients (11 with repaired TOF and 5 with repaired PS) completed the protocol with adequate data for analysis. The median age [range] was 35 [19–46] years, BMI was 26 ± 5 kg/m^2^ (mean ± SD), 50% were women and 75% were in NYHA class I. Right ventricular end diastolic volume index for the cohort was 157 ± 33 mL/m^2^, end systolic volume index was 93 ± 20 mL/m^2^ and right ventricular ejection fraction was 40 ± 6%. Baseline pulmonary regurgitant volume was 45 ± 25 mL/beat and regurgitant fraction was 35 ± 16%. During administration of iNO, regurgitant volume was reduced by an average of 6 ± 9% (p=0.01) and regurgitant fraction was reduced by an average of 5 ± 8% (p=0.02). No significant changes were observed in ventricular indices for either the left or right ventricle.

**Conclusion:**

iNO was successfully administered during CMR acquisition and appears to reduce regurgitant fraction in patients with at least moderate PI suggesting a potential role for selective pulmonary vasodilator therapy in these patients.

**Trials registration:**

ClinicalTrials.gov, NCT00543933

## Background

Pulmonic valve insufficiency (PI) is a well-defined problem following primary surgical repair of tetralogy of Fallot (TOF) or valvar pulmonic stenosis (PS). The early experience with surgical repair suggested that residual outflow tract obstruction led to poor short-term outcomes [1]. Combined with the notion that PI was inconsequential, this led to an era where complete relief of obstruction was emphasized, often at the expense of valve integrity [2]. Longstanding PI is well tolerated during childhood but leads to progressive right ventricular (RV) enlargement, right and left ventricular dysfunction, arrhythmia and sudden cardiac death during adult years [3].

There are no medical therapies currently available for this growing population and the only management strategy has been valve repair or replacement. Conservative management has been recommended for patients with PI until evidence of RV dilation or symptoms of heart failure develop. Early valve replacement increases the chance of repeat open heart surgery for graft failure which occurs approximately every 10 years, whereas delayed valve replacement may not reverse structural changes in the right and left ventricle that have already occurred. The optimal timing of pulmonary valve replacement remains controversial; although recent cardiovascular magnetic resonance (CMR) data suggests that when the right ventricular end diastolic volume index is >170 mL/m^2^ and end systolic volume index is >85 mL/m^2^, there is no decrease in ventricular size after valve surgery [4-6].

Symptoms associated with long standing PI mirror those associated with chronic aortic insufficiency (AI). Afterload reduction for AI appears to improve the hemodynamic milieu and may prolong the timing to valve replacement surgery [7,8]. Despite proven benefit, systemic afterload reduction has failed to be a panacea for chronic AI, likely related to the considerable diastolic gradient that needs to be overcome [9]. In AI, the diastolic pressure gradient across the valve can range from 30-70 mmHg [10]. As a result, lowering the systemic blood pressure by only a few millimeters of mercury is unlikely to affect the overall degree of regurgitation. PI is driven by a far lower diastolic gradient (<10 mmHg) and may be easier to influence with medications that lower pulmonary vascular resistance [11]. This study assessed the ability of inhaled nitric oxide, a selective pulmonary vasodilator with a rapid onset of action, to acutely decrease pulmonary regurgitant fraction by improving afterload mismatch as measured using CMR, the established gold standard for measuring pulmonary regurgitant fraction.

## Methods

### Study population

This was a single-center prospective study conducted at the Cleveland Clinic with approval from the Institutional Review Board and registered with the National Library of Medicine (NCT00543933). Subjects underwent appropriate consent with strict adherence to Health Insurance Portability and Accountability Act regulations. Consecutive individuals presenting for a clinically indicated CMR at the Cleveland Clinic with evidence of PI by echocardiography and a history suggestive of repaired TOF or PS were approached for enrollment. Patients who had a right ventricular-to-pulmonary artery conduit, underwent late pulmonary valve repair or replacement for PI, had residual shunt lesions, had residual pulmonary valvular/subvalvular (mean gradient >30 mmHg by echocardiography) or branch pulmonary stenosis, or evidence of pulmonary hypertension were excluded. Individuals were also excluded if contraindications to inhaled nitric oxide or CMR existed.

After consent was obtained, each individual had a brief assessment including collection of demographics, medical history and physical examination including vital signs and assessment of New York Heart Association (NYHA) functional class. Detailed medical history was taken directly from chart review. Elements of past surgical history including history of systemic to pulmonary shunts or other palliative shunts, type of repair performed, presence of right ventricular outflow tract (RVOT) aneurysm and presence of residual pulmonary stenosis were obtained.

### Cardiovascular magnetic resonance

CMR was performed at 1.5 Tesla (Magnetom Avanto; Siemens Medical Systems, Erlangen, Germany) with a multi-element phased-array surface coil. Studies were performed with patients in the supine position using a phased-array surface coil as a receiver and retrospective electrocardiographic gating. Images were obtained during end-expiratory breath holds preceded by brief hyperventilation. CMR images were downloaded to an off-line workstation (Leonardo; Siemens Medical Solutions) for analysis.

After obtaining standard localizer views and contiguous short-axis cine views covering both ventricles (encompassing the entire left and right ventricles from the base to the apex), two double oblique views oriented along the main axis of the pulmonary trunk and the aortic root were acquired with a standard steady-state free precession (SSFP) cine magnetic resonance sequence for the positioning of phase-contrast magnetic resonance images and to ensure that the imaging plane remained in the proper position throughout the whole cardiac cycle. Cine steady state free-precision image parameters were: TR (repetition time)/TE (echo time) 3.2 ms/1.6ms, flip angle (70°), an 8-mm section thickness with a gap of 2 mm (10–12 short axis images in total), matrix of 256 × 166 (typical), minimal field of view (typical), typical spatial resolution of 1.7 × 1.4 mm, acquired temporal resolution of 33–45 ms, 20–25 reconstructed cardiac phases (reconstructed by using view sharing when required), and a bandwidth of 900–1000 Hz/pixel. Endocardial contours in end systole and end diastole were manually traced to derive ventricular volumes using the disc-summation technique. The following measurements were then obtained for both the right and left ventricles: end diastolic volume, end systolic volume, stroke volume and ejection fraction.

Phase-contrast velocity-encoded CMR was acquired with a segmented fast gradient-echo sequence, with velocity encoding perpendicular to the imaging plane and a predefined upper velocity limit of 100 cm/s. If aliasing was noted, the velocity was progressively raised in 50-cm/s steps until aliasing disappeared. Imaging parameters included: TR/TE 7.5 ms/3.1 ms; flip angle, 15°; section thickness, 6 mm; field of view, 320–380 × 240–300 mm; matrix, 256 × 96 (typical in-plane resolution, 2.7 × 1.4 mm); number of signals acquired, one; temporal resolution, 35–45 ms; number of reconstructed cardiac phases, 25; bandwidth, 260 Hz/pixel. The typical breath-hold time for phase-contrast velocity-encoded sequences ranged from 15–20 seconds. If obvious breathing artifacts were noted, repeated acquisitions were attempted. Phase-contrast images were post-processed using commercially available software (Argus; Siemens Medical Solutions). The heart rate during CMR was recorded from the acquired images. The contours of the main pulmonary artery and aorta were automatically traced, with manual correction, simultaneously on magnitude and velocity-map images of all 25 reconstructed phases. Velocity in each of the voxels included within the contour was calculated and integrated over area and time to obtain the following parameters: average velocity, average flow, minimum area, maximum area, forward volume and reverse volume. Regurgitant fraction was calculated as reverse volume/forward volume. Tricuspid regurgitation was calculated as right ventricular stroke volume – pulmonary artery forward flow. Qp:Qs ratio was calculated as net forward pulmonary artery flow/net forward aortic flow.

### Inhaled nitric oxide

The standard delivery method for iNO has been well described [12]. Medical grade nitric oxide gas (INOMAX®, IKARIA, Clinton, NJ) is typically delivered via the INOvent® delivery system (IKARIA, Clinton, NJ) from source tanks, and flushed through to the patient using supplemental O_2_ flow at 5 L/min. The dose of iNO (ppm) is controlled by the INOvent regulator and confirmed by the electrochemical analyzer incorporated into the INOvent. Concentrations of NO (ppm), NO_2_ (ppm) and O_2_ (%) are continuously reported to the INOvent display from the analyzer. The magnetic field created by the CMR suite required modification to the standard delivery technique which has been previously described by our group [13].

Once baseline imaging was completed, iNO was continuously administered to the patient. After 5 minutes with the patient breathing inhaled nitric oxide to ensure steady state conditions, imaging was repeated. All volumetric measurements were indexed to body surface area using the DuBois method [14]. Two independent readers interpreted each study and the mean value of each measurement was used for analysis. The acquisition technique prevented blinding readers to the study sequence. A follow-up phone call was placed with each subject 1 day following the study to monitor for any adverse events.

### Statistics

Sample size was estimated from a CMR study measuring change in aortic regurgitant fraction in response to intravenous felodipine in 16 asymptomatic patients with aortic insufficiency [15]. Based on this study we anticipated that 22 patients would be required for a 90% power to detect a 6% decrease in regurgitation change with an estimated standard deviation of 6% using a significance of 0.05 accounting for a dropout rate of 40% in paired analysis. PASS 2005 (NCSS, 2005) was used for sample size determination.

Data are presented as mean ± standard deviation. Linear regression was used to test univariate associations. CMR volumetric and flow measurements were compared using paired t-tests to assess the effect of intervention between baseline measurements with repeated values obtained during the administration of iNO. Significance was assumed to be p<0.05 for all tests. All analyses were performed using JMP Pro 9 (SAS, 2010).

## Results

### Study population

**Figure 1:**
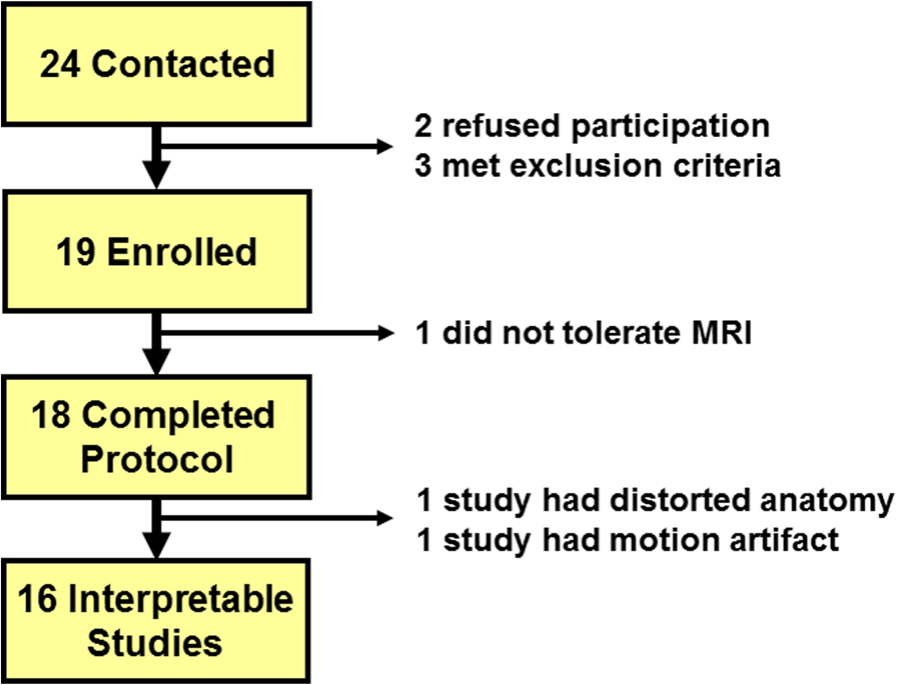
**Patient flow diagram.** Twenty four potential subjects were contacted and 18 completed the imaging protocol. One of these patients had motion artifact and another had significantly distorted anatomy preventing adequate measurements.

Twenty four patients were contacted regarding the study after initial screening (Figure 1). Two patients refused participation, 3 met exclusion criteria, 1 was unable to tolerate the CMR due to clostrophobia and 2 patients had incomplete CMR data available and were excluded from analysis. The final cohort included 16 patients (Table 1).

**Table 1:** Demographics and clinical characteristics

	**All patients**
	n=16
Demographics:	
Females	8 (50)
Age (years)	33.6 ± 8.8
Age at complete repair (years)	5.7 ± 6.8
Body mass index (kg/m^2^)	25.8 ± 5.4
Clinical Features:	
Repaired tetralogy of Fallot	11 (69)
Repaired pulmonary stenosis	5 (31)
Symptoms*	11 (69)
NYHA Class ≥ II	4 (25)
Echocardiography:	
Pulmonary insufficiency ≥ moderate	13 (87)
Tricuspid regurgitation ≤ mild	15 (93)
Right ventricular systolic pressure (mmHg)	35.8 ± 13.6
Electrocardiography	
QRS duration (ms)	139.5 ± 28.2

*Includes shortness of breath, dyspnea on exertion or palpitations.

NYHA Class: New York Heart Association Functional Class.

Data presented as mean ± standard deviation or number (%).

Half the cohort was female (n=8, 50%) and the median age [interquartile range] was 34.8 [23.4 – 40.8] years. The average body mass index of the cohort was 25.8 ± 5.4 kg/m^2^ and most patients were in NYHA functional class I at the time of the study (n=12, 75%). Eleven patients reported intermittent symptoms (exercise intolerance, shortness of breath or palpitations) at the time of evaluation. Two patients had systemic hypertension and no patients were taking medications for or had symptoms of coronary artery disease.

Eleven patients (69%) had a history of TOF, while five (31%) had a history of isolated PS. The median age [interquartile range] for complete surgical repair among TOF and PS patients was 3.7 [1.8 – 4.6] years. Most patients had echocardiographic evidence of at least moderate PI (n=13, 87%) and all but one had mild tricuspid valve regurgitation or less (n=15, 93%). Mean estimated right ventricular systolic pressure by echocardiography was 35.8 ± 13.6 mmHg for the cohort. The mean QRS duration was 139.5 ± 28.2 ms on electrocardiography.

### Volumetric analysis

Volumetric results at baseline and with iNO are shown in Table 2. At baseline, right ventricular end diastolic volume index was 156.9 ± 32.7 mL/m^2^, right ventricular end systolic volume index was 93.0 ± 19.6 mL/m^2^, right ventricular stroke volume index was 63.9 ± 17.8 mL/m^2^ and right ventricular ejection fraction was 40.5 ± 6.2%. All parameters remained unchanged during administration of iNO (p=0.95, p=0.39, p=0.21 and p=0.12 respectively).

**Table 2:** Volumetric measurements

	**Baseline**	**iNO**	**% Change**	p-value*
Right Ventricle (n=16)				
End diastolic volume index (mL/m^2^)	156.9 ± 32.7	156.7 ± 31.0	0.1 ± 7.1	0.95
End systolic volume index (mL/m^2^)	93.0 ± 19.6	90.6 ± 20.5	−2.3 ± 10.9	0.39
Stroke volume index (mL/m^2^)	63.9 ± 17.8	66.1 ± 17.8	4.2 ± 11.0	0.21
Ejection fraction %	40.5 ± 6.2	42.1 ± 6.8	4.4 ± 10.7	0.12
Left Ventricle (n=16)				
End diastolic volume index (mL/m^2^)	83.0 ± 13.6	84.6 ± 13.2	2.2 ± 5.7	0.15
End systolic volume index (mL/m^2^)	38.5 ± 8.7	39.5 ± 8.4	3.1 ± 5.8	0.11
Stroke volume index (mL/m^2^)	44.5 ± 7.7	45.1 ± 8.1	1.7 ± 11.5	0.65
Ejection fraction %	53.8 ± 5.2	53.3 ± 5.8	−1.0 ± 0.7	0.63

*By paired t-test between baseline values and iNO values.

At baseline, left ventricular end diastolic volume index was 83.0 ± 13.6 mL/m^2^, left ventricular end systolic volume was 38.5 ± 8.7 mL/m^2^, left ventricular stroke volume was 44.5 ± 7.7 mL/m^2^ and ejection fraction was 53.8 ± 5.2%. All parameters remained unchanged during iNO administration (p=0.15, p=0.11, p=0.65 and p=0.63 respectively).

### Flow analysis

**Figure 2:**
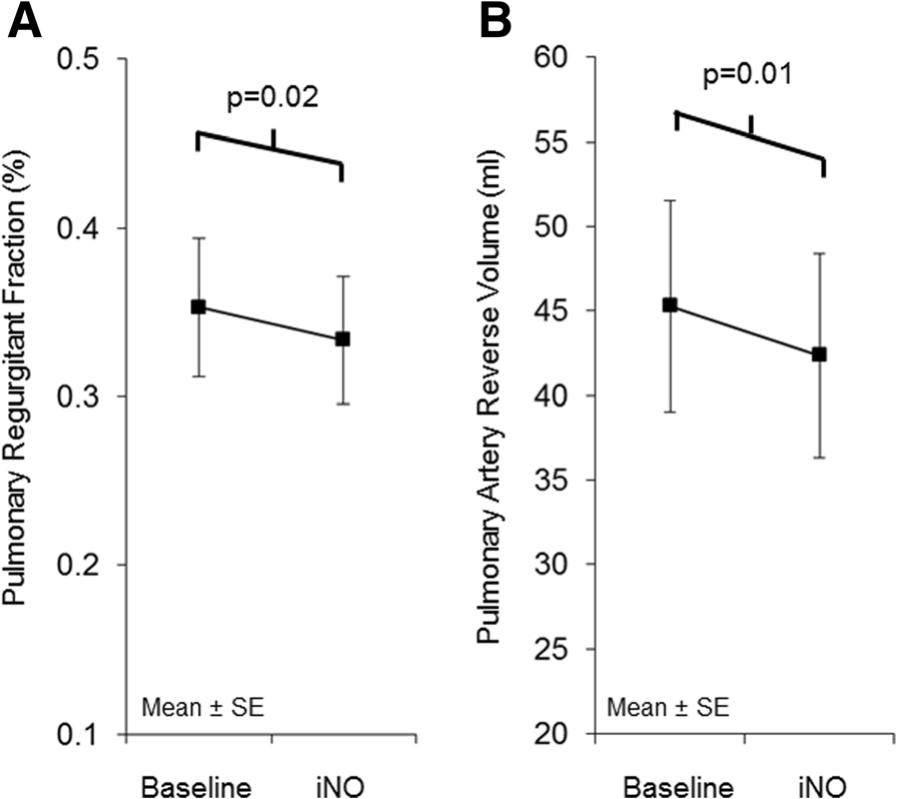
**Effect of iNO in matched paired analysis (n=16). (A)** Pulmonary regurgitant fraction and **(B)** pulmonary artery reverse volume at baseline and during administration of 40 ppm iNO.

**Figure 3:**
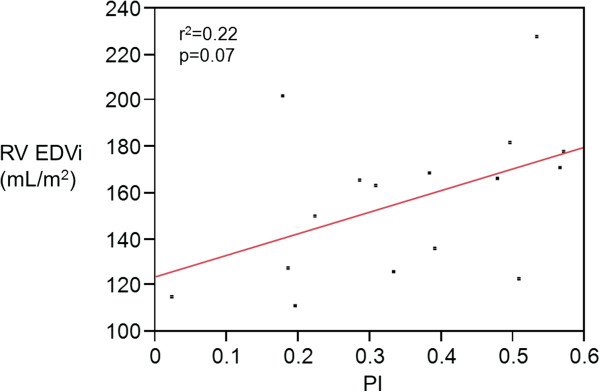
**Right ventricular end diastolic volume index (RV EDVi) as a function of pulmonary insufficiency (PI) (n=16).** RV EDVi has been shown to correlate with PI in large scale studies. We observed a similar relationship but with a low r^2^ value.

Pulmonary artery flow parameters are displayed in Table 3. Average velocity in the pulmonary artery was 15.3 ± 8.1 m/s at baseline and did not change with iNO (p=0.17). Average flow index was 2.7 ± 0.7 L/min/m^2^ at baseline and did not change with iNO either (p=0.13). The forward volume across the pulmonary artery was similar before and after iNO administration (123.5 ± 26.8 vs. 122.7 ± 31.6 mL/beat/m^2^, p=0.75) but regurgitant fraction (35.3 ± 16.4 to 33.3 ± 15.1%, p=0.02, Figure 2A) and reverse volume decreased with iNO (45.3 ± 25.0 vs. 42.4 ± 24.1 mL/beat/m^2^, p=0.01, Figure 2B). Right ventricular end diastolic volume index appeared to be weakly correlated to the magnitude of PI (r^2^=0.22, p=0.07, Figure 3), but PI did not differ according to sex (p=0.34) or diagnosis (p=0.70).

**Table 3:** Flow measurements

	**Baseline**	**iNO**	**% Change**	p-value*
Pulmonary Artery (n=16)				
Average velocity (m/s)	15.3 ± 8.1	15.9 ± 8.9	2.7 ± 12.4	0.17
Average flow index (L/min/m^2^)	2.7 ± 0.7	2.6 ± 0.8	−2.3 ± 10.2	0.13
Forward volume (mL/beat)	123.5 ± 26.8	122.7 ± 31.6	−1.1 ± 8.6	0.75
Reverse volume (mL/beat)	45.3 ± 25.0	42.4 ± 24.1	−6.5 ± 8.6	0.01
Regurgitant fraction %	35.3 ± 16.4	33.3 ± 15.1	−5.2 ± 7.8	0.02
Aorta (n=11)				
Average velocity (m/s)	14.1 ± 6.2	13.4 ± 6.3	−4.8 ± 8.0	0.10
Average flow index (L/min/m^2^)	2.9 ± 0.8	3.0 ± 0.9	5.0 ± 13.0	0.71
Forward volume (mL/beat)	74.8 ± 17.2	71.3 ± 18.8	−6.3 ± 9.7	0.05
Reverse volume (mL/beat)	3.2 ± 4.4	3.1 ± 5.1	−28.4 ± 43.4^†^	0.73
Regurgitant fraction %	4.5 ± 6.4	4.2 ± 5.4	−23.1 ± 45.1^†^	0.51

*By paired t-test between baseline values and iNO values.

^†^Outliers excluded.

No significant changes in aortic flow parameters were detected during administration of iNO (Table 3). Average velocity was 14.1 ± 6.2 m/s, average flow index was 2.9 ± 0.8 L/min/m^2^, forward volume was 74.8 ± 17.2 mL/beat and reverse volume was 3.2 ± 4.4 mL/beat with no significant changes during iNO administration (p=0.10, p=0.71, p=0.052 and p=0.73, respectively). Aortic regurgitant fraction was minimal at baseline (4.5 ± 6.4) and did not change during iNO administration (p=0.51). Large % changes were observed in reverse volume and regurgitant volume with iNO administration but the absolute changes were minimal. Outliers in these fields were removed for improved data clarity.

**Figure 4:**
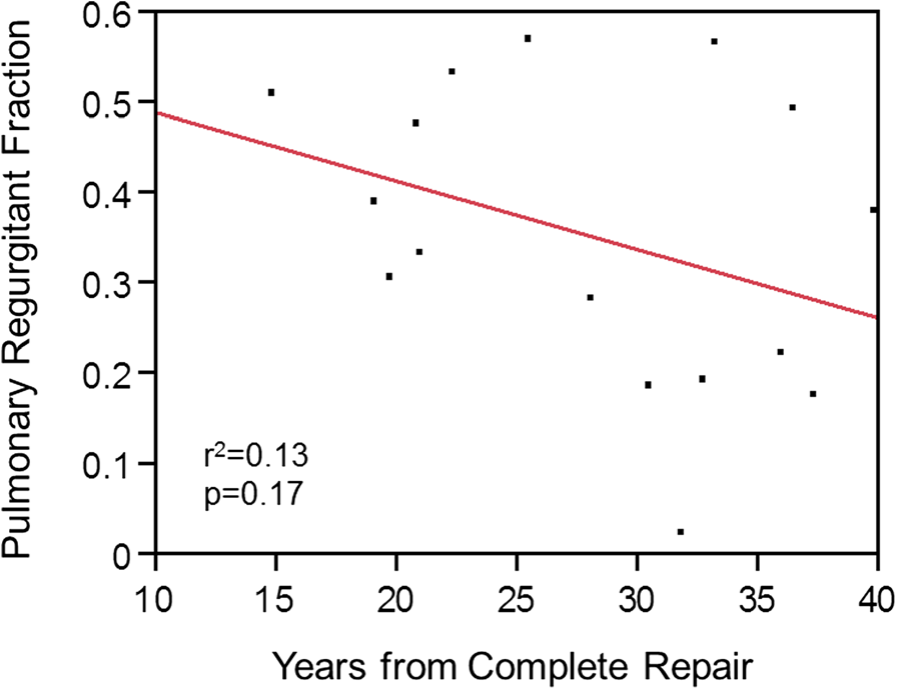
**Pulmonary Insufficiency (PI) as a function of time from complete repair (n=16).** PI is known to be a progressive disease and our observation of the contrary could reflect referral bias as most patients enrolled were referred for evaluation for symptoms.

The length of time from complete surgical repair to enrollment in the study did not appear to be associated with NYHA function class (r^2^=0.01, p=0.74), right ventricular end diastolic volume (r^2^=0.03, p=0.54), right ventricular ejection fraction (r^2^=0.18, p=0.10), QRS duration (r^2^=0.18, p=0.22) or the degree of pulmonary insufficiency (r^2^=0.13, p=0.17, Figure 4). Age at complete repair did not appear to be associated with these variables either. At baseline the mean Qp:Qs ratio was 1.13 and mean tricuspid regurgitation was 6.7%.

## Discussion

This study is the first to show that inhaled nitric oxide may be safely and effectively delivered inside a CMR suite in a cohort of adult patients with congenital heart disease and demonstrates that inhaled nitric oxide acutely reduces the severity of PI as measured by CMR which is the key determinant in late outcomes following repaired TOF and PS. It has been well established that patients with PI and increased afterload develop early right ventricular failure [16-18]. Furthermore, Chaturvedi and colleagues demonstrated a linear relationship between right ventricular afterload and the magnitude of PI in patients undergoing balloon valvuloplasty of distal stenosis [19]. It is likely that iNO induced a decrease in pulmonary artery pressure, similar to the effect seen in patients with pulmonary arterial hypertension, which resulted in improved right ventricular afterload mismatch and decreased PI.

While significant, the absolute reductions in pulmonary regurgitant volume and regurgitant fraction were modest and the effect size did not produce significant changes in ventricular indices. The hemodynamic effects of iNO on PI might have been underestimated for a number of reasons. The half-life of nitric oxide is shorter than the typical breath hold sequence and variability likely exists in the length of breath holds between patients. The severity of PI also varied among the cohort. Two patients with echocardiographic evidence of at least moderate PI were enrolled in the study but had only minimal PI on CMR which reduced the observed effect size. Finally, it has been recently shown that pulmonary regurgitant fraction is significantly smaller when measured in end expiration as compared with other techniques [20]

Right ventricular end diastolic volume and the magnitude of PI appeared to be related in this study (Figure 3). This relationship has been observed in other cohorts and represents a compensated stage of chronic volume overload [21-24]. No patient had more than moderate systolic dysfunction on enrollment, so it is likely that our cohort was well compensated. Despite this, the majority of patients had some degree of symptoms suggestive of right heart failure; and on follow up, 10 of 16 patients (63%) were noted to have undergone pulmonary valve replacement within 1 year of study enrollment.

No discernible relationship was observed between right ventricular end diastolic volume or PI and time from complete repair, which is contrary to the perceived natural history of chronic volume overload [25]. This may be indicative of changes in the operative procedure toward more valve sparing procedures over time resulting from more effective surgical visualization in recent years, though an alternative explanation may be selection bias. A large proportion of patients over 30 years out from complete repair would have previously developed ventricular dysfunction necessitating intervention that precluded them from enrollment in this study. There is a complex interplay between PI and compensatory mechanisms of the right ventricle, and studies have demonstrated that restrictive right ventricular physiology is a risk factor for poor outcomes early after TOF repair, but may be protective of ventricular dilation late after repair. The patients who are more than 30 years from complete repair enrolled in this study potentially represent a population who are more resistant to ventricular remodeling, perhaps due to the presence of restrictive physiology early in life.

The population chosen for this study was selected to minimize confounding physiology. Patients with residual shunt lesions or with prior surgical reintervention on the RVOT were excluded when this was recognized. Patients with residual RVOT or branch pulmonary artery stenosis were also excluded, because it is unknown how these lesions might affect the magnitude of PI or influence hemodynamic changes observed with iNO.

Sustained pulmonary vasodilation with a longer acting agent may possibly result in a greater effect size and more consistent results. A recent study by Babu-Narayan and colleagues, however, demonstrated no change in severity of PI after 6 months of oral ramipril therapy in a similar population of patients [26]. Angiotensin converting enzyme inhibitors (ACEi) have myriad effects on the cardiovascular system including vasodilation, afterload reduction, ventricular remodeling and neuro-hormonal modulation. A more specific drug targeting pulmonary vasodilation, such as an oral phosphodiesterase-5 inhibitor, might have a more potent vasodilatory effect and result in a larger and more sustained reduction in PI than either iNO or oral ACEi.

There are limitations to this study that must be considered when assessing applicability. The study was carried out at a single quaternary care medical center and is therefore subject to referral bias. The majority of patients carried the diagnosis of TOF, though 5 had only PS as their initial lesion. We did not have the power to discern significant differences between these 2 populations, though differences potentially exist. Furthermore, the degree of PI varied considerably across the cohort, with 2 patients having almost no PI on CMR. This study also lacks precise details of the primary repair and peri-operative management which have been suggested to influence late outcomes [27-30]. Despite these limitations, this study suggests a possible role for selective pulmonary vasodilation as a target for therapeutic development in patients with PI and lays the foundation for future studies.

## Conclusion

This study demonstrates that inhaled nitric oxide may be delivered within a CMR suite and serves as a proof-of-concept for pulmonary vasodilator therapy in patients with PI. Long term pulmonary vasodilation with oral agents such as phosphodiesterase-5 inhibitors may have similar effects to those seen following pulmonary valve repair or replacement with potentially less risk. Further studies are necessary to evaluate the effect of these agents on this complex disorder, particularly given the ever growing patient population.

## Abbreviations

PI: Pulmonic insufficiency; TOF: Tetralogy of Fallot; PS: Pulmonic stenosis; RV: Right ventricle; AI: Aortic insufficiency; CMR: Cardiovascular magnetic resonance; NYHA: New York heart association; RVOT: Right ventricular outflow tract; SSFP: Steady-state free precession; iNO: Inhaled nitric oxide; ACEi: Angiotensin converting enzyme inhibitors.

## Competing interests

Dr. Krasuski serves on the scientific advisory board for Ventripoint, as a consultant to Actelion and on the speakers bureaus of Actelion and Roche. The other authors report no industry relationships.

## Authors’ contributions

SH participated in the study design, subject recruitment, data collection, data analysis, manuscript drafting, and critical review of the manuscript. GD participated in subject recruitment, data analysis, and critical review of the manuscript. YK participated in acquiring study funding, study design, subject recruitment, and critical review of the manuscript. SF participated in the study design, study coordination, data collection, data analysis, and critical review of the manuscript. VK participated in data collection, data analysis, and critical review of the manuscript. JA participated in data analysis and critical review of the manuscript. EW participated in data analysis, statistical analysis, and critical review of the manuscript. TB participated in data collection, data analysis, and critical review of the manuscript. MB participated in data collection, data analysis and critical review of the manuscript. RS provided technical support, provided study coordination, data analysis, and critical review of the manuscript. RK conceived of the study, participated in study design, subject recruitment, data analysis, critical review of the manuscript and overall study coordination. All authors read and approved the final manuscript.
